# Supplementary material for: Understanding Cell Model Characteristics—RNA Expression Profiling in Primary and Immortalized Human Mesothelial Cells, and in Human Vein and Microvascular Endothelial Cells
Source: Cells. 2022 Oct 5;11(19):3133. doi: 10.3390/cells11193133 (PMC9563025; doi:10.3390/cells11193133)
Supplement: Supplementary file 1 [file cells-11-03133-s001.zip › Supplemental figures_revised.pdf]

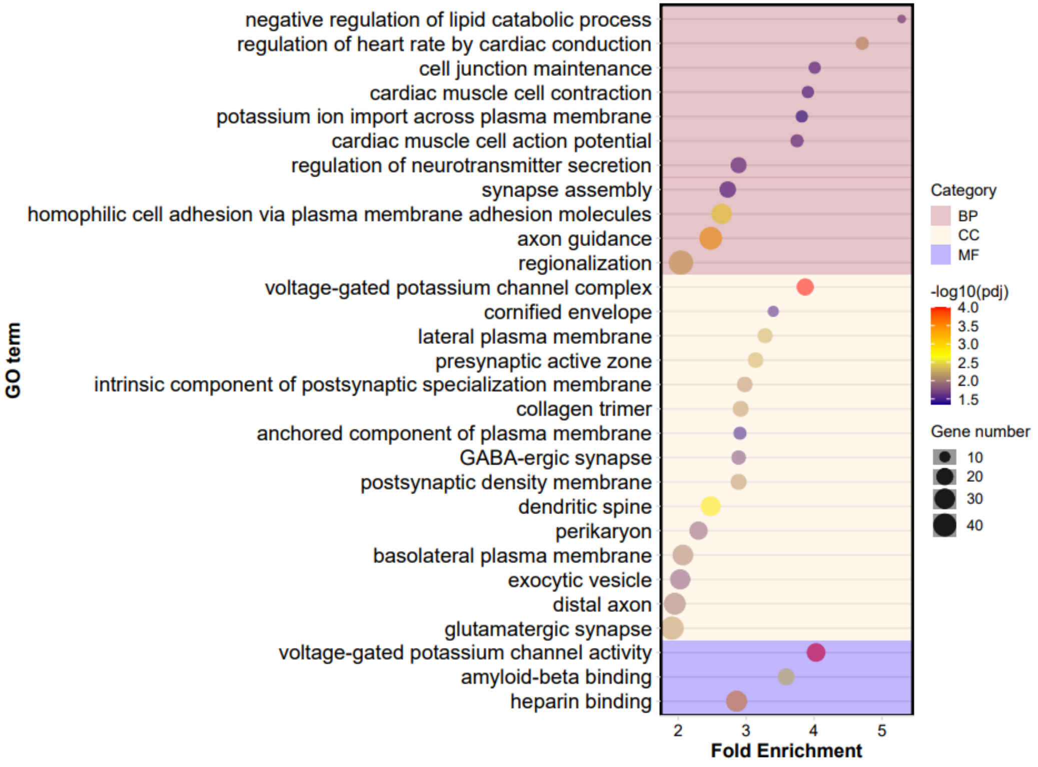


**Figure S1. Gene Ontology (GO) term enrichment analysis of 1000 mesothelial cell specific genes.** Red color represents significantly regulated biological process (BP), green cellular component (CC), blue molecular function (MF) identified by Panther Gene Ontology Enrichment Analysis (FDR <0.05).

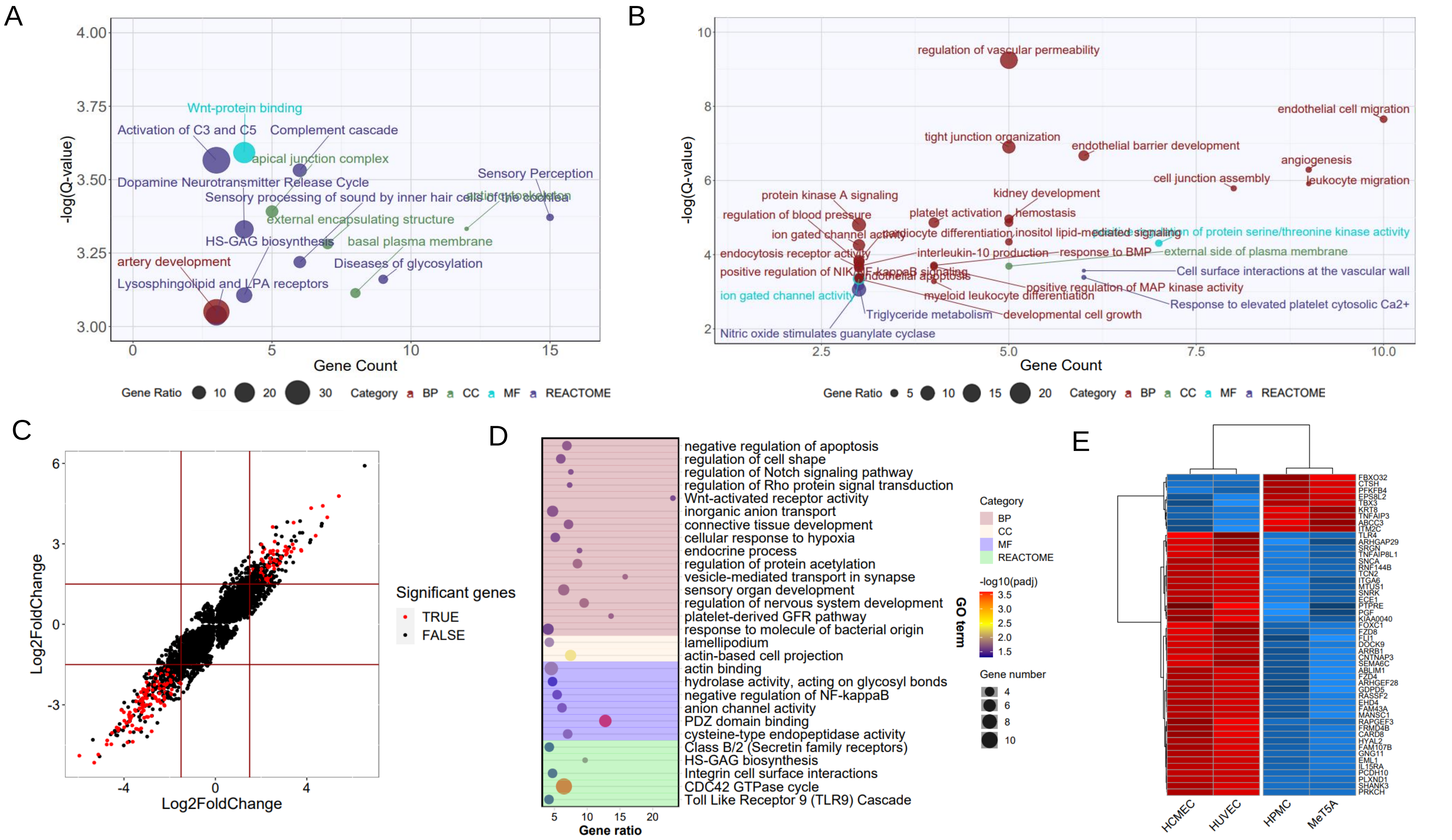


**Figure S2. Gene Ontology (GO) term and Reactome pathway enrichment analysis of mesothelial and endothelial genes.** A) Enrichment analysis of 393 genes found in both mesothelial cell lines (HPMC and MeT-5A) B) Enrichment analysis of 235 genes found in both endothelial cell lines (HUVEC and HCMEC). Red color represents significantly regulated biological processes (BP), green cellular components (CC), light blue molecular functions (MF) and purple Reactome pathways as identified by ClueGO (FDR <0.05) C) Volcano plot showing 174 genes out of the genes shared between mesothelial and endothelial cells with significantly different expression and D) enrichment analysis of the 174 genes depicted in C). A heatmap of the 50 genes with highest difference in expression levels between mesothelial and endothelial cells is given in E).

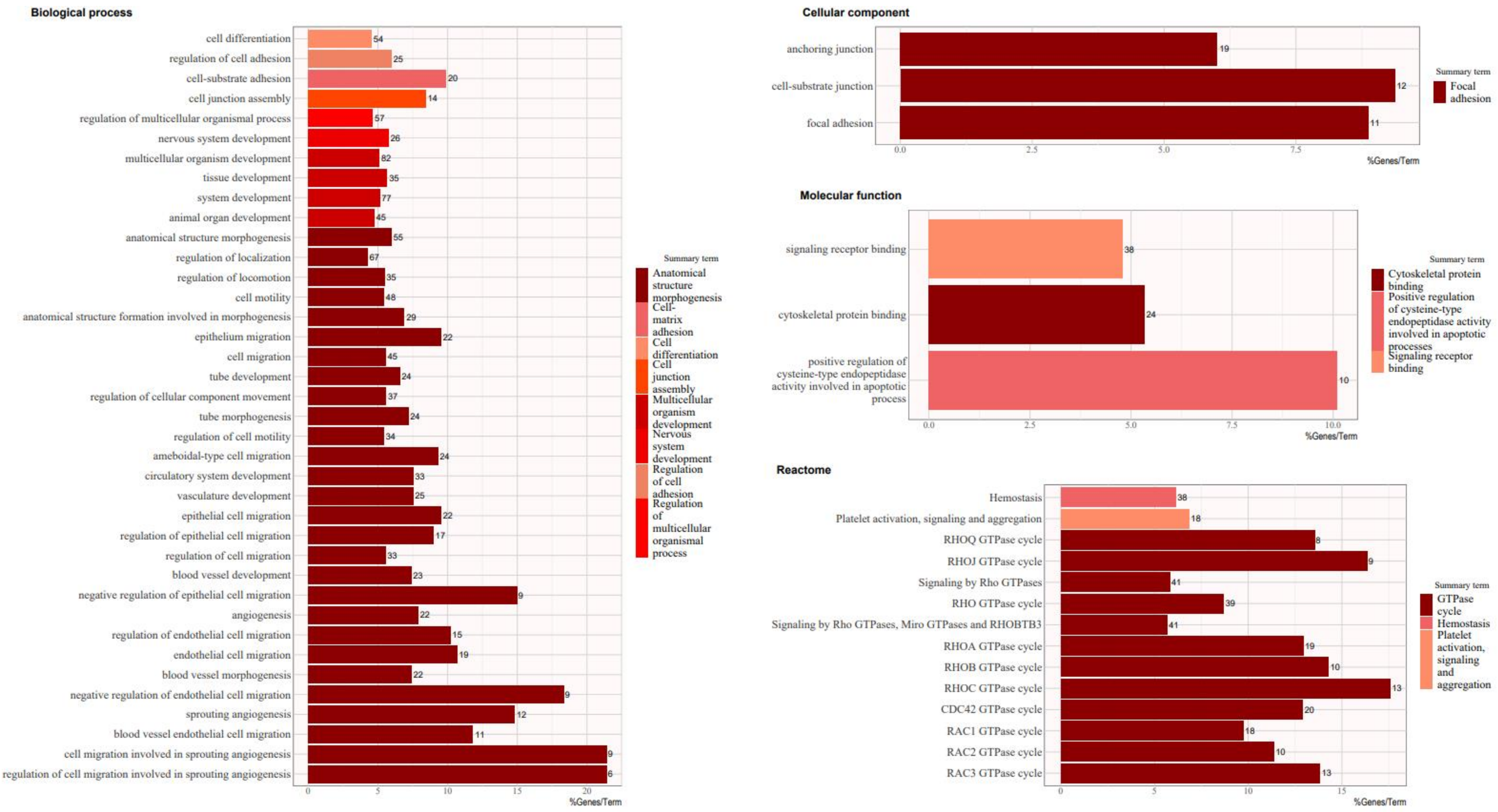


**Figure S3. Enrichment analysis of cluster 3 (Circulatory system development)** as given in Figure 6 which compares transcriptional profiles in 4 studied cell lines. Bar plots show gene sets and pathways of cluster 3. These were analyzed by biological process-, cellular component-, molecular function- gene ontology (GO) enrichment analysis and using Reactome pathway analysis. All significantly enriched terms are clustered into parental summary term by ClueGO (Cytoscape app) are given.

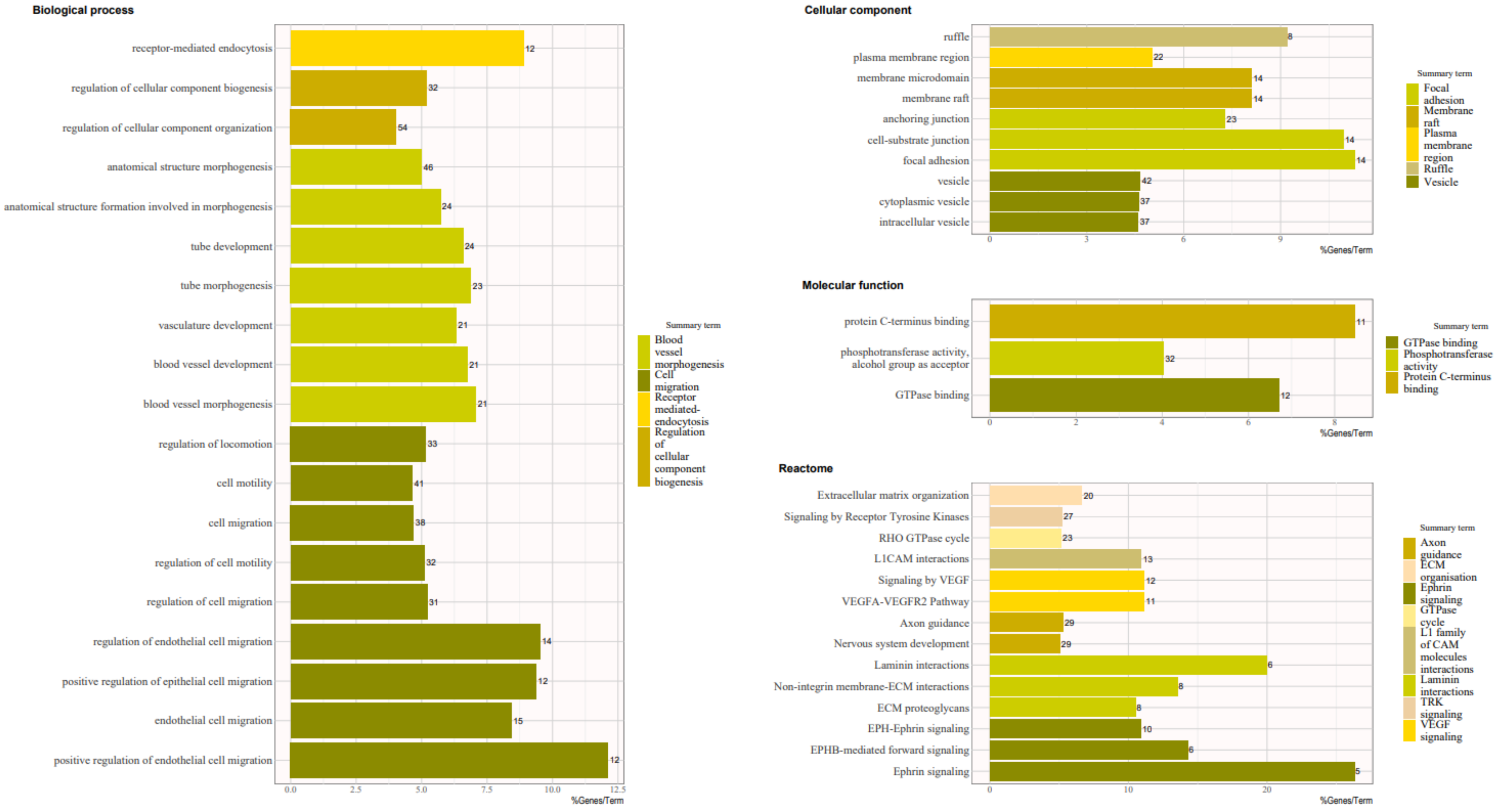


**Figure S4. Enrichment analysis of cluster 4 (Cell migration)** as given in Figure 6 which compares transcriptional profiles in 4 studied cell lines. Bar plots show gene sets and pathways of cluster 4. These were analyzed by biological process-, cellular component-, molecular function- gene ontology (GO) enrichment analysis and using Reactome pathway analysis. All significantly enriched terms as clustered into parental summary term by ClueGO (Cytoscape app) are given.

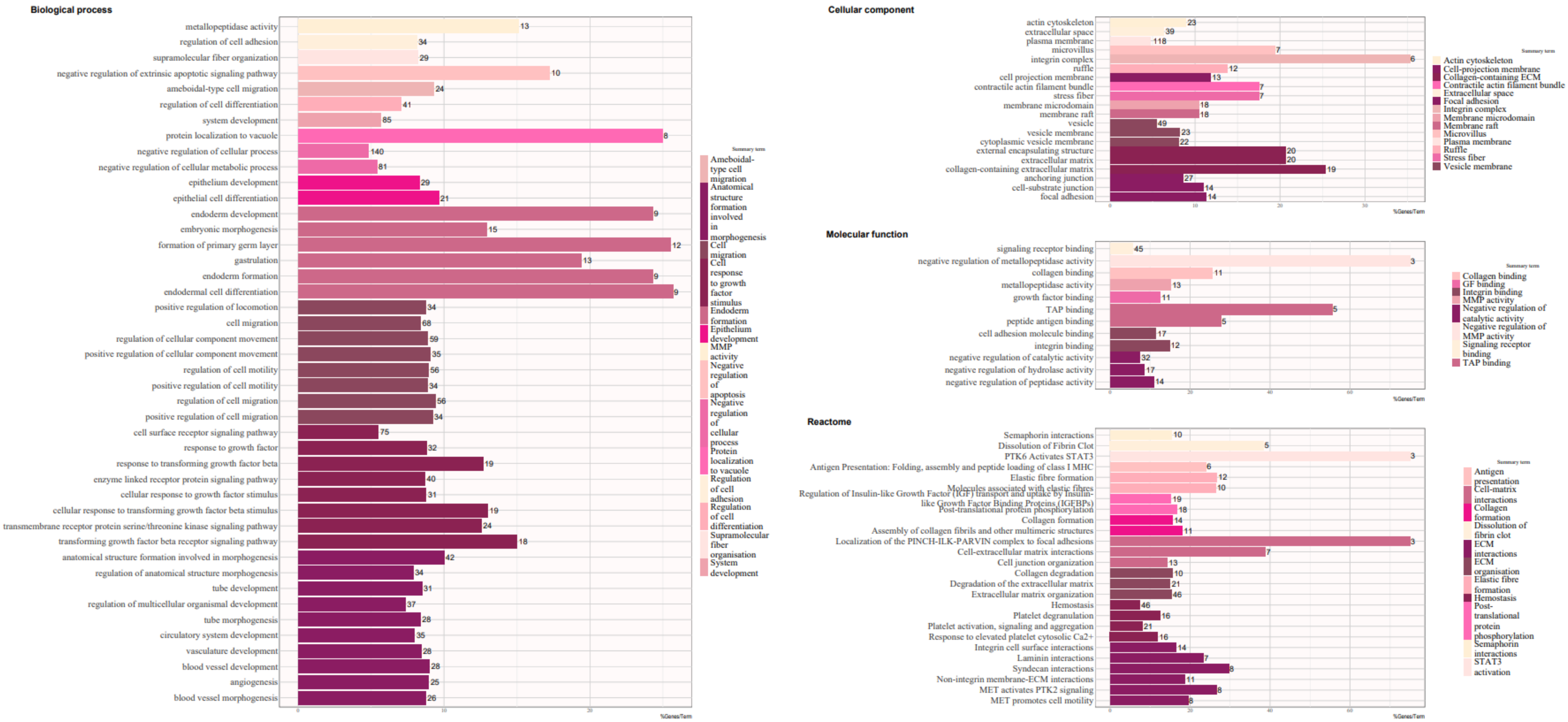


**Figure S5. Enrichment analysis of cluster 2 (Regulation of vasculature development)** as given in Figure 6 which compares transcriptional profiles in 4 studied cell lines. Bar plots show gene sets and pathways of cluster 2. These were analyzed by biological process-, cellular component-, molecular function- gene ontology (GO) enrichment analysis and using Reactome pathway analysis. All significantly enriched terms as clustered into parental summary term by ClueGO (Cytoscape app) are given.

5A

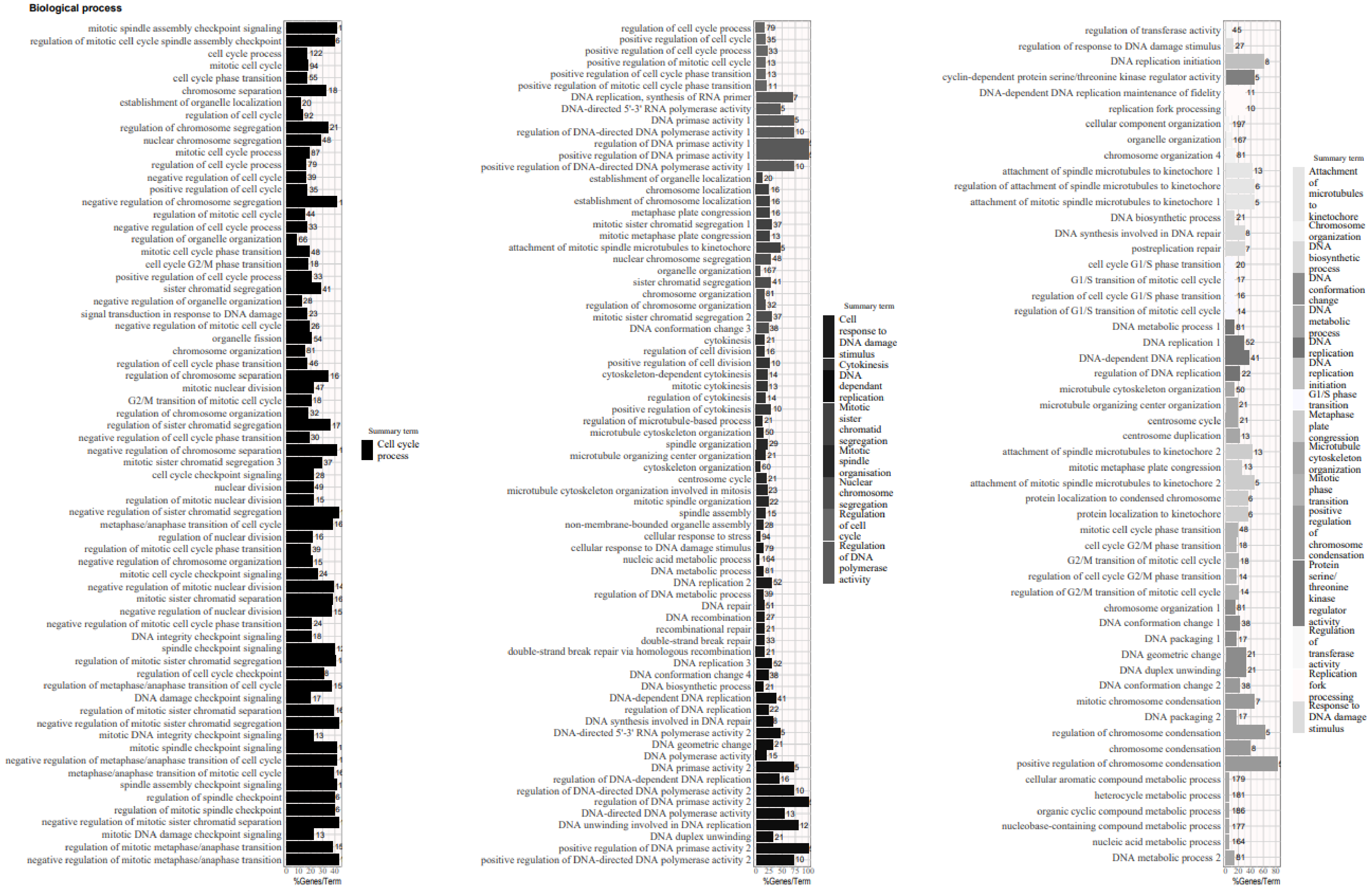

Biological process
mitotic spindle assembly checkpoint signaling
regulation of mitotic cell cycle spindle assembly checkpoint
cell cycle process
mitotic cell cycle
cell cycle phase transition
chromosome separation
establishment of organelle localization
regulation of cell cycle
regulation of chromosome segregation
nuclear chromosome segregation
mitotic cell cycle process
regulation of cell cycle process
negative regulation of cell cycle
positive regulation of cell cycle
negative regulation of chromosome segregation
regulation of mitotic cell cycle
negative regulation of cell cycle process
regulation of organelle organization
mitotic cell cycle phase transition
cell cycle G2/M phase transition
positive regulation of cell cycle process
sister chromatid segregation
negative regulation of organelle organization
signal transduction in response to DNA damage
negative regulation of mitotic cell cycle
organelle fission
chromosome organization
regulation of cell cycle phase transition
regulation of chromosome separation
mitotic nuclear division
G2/M transition of mitotic cell cycle
regulation of chromosome organization
regulation of sister chromatid segregation
negative regulation of cell cycle phase transition
negative regulation of chromosome separation
mitotic sister chromatid segregation 3
cell cycle checkpoint signaling
nuclear division
regulation of mitotic nuclear division
negative regulation of sister chromatid segregation
metaphase/anaphase transition of cell cycle
regulation of nuclear division
regulation of mitotic cell cycle phase transition
negative regulation of chromosome organization
mitotic cell cycle checkpoint signaling
negative regulation of mitotic nuclear division
mitotic sister chromatid separation
negative regulation of nuclear division
negative regulation of mitotic cell cycle phase transition
DNA integrity checkpoint signaling
spindle checkpoint signaling
regulation of mitotic sister chromatid segregation
regulation of cell cycle checkpoint
regulation of metaphase/anaphase transition of cell cycle
DNA damage checkpoint signaling
regulation of mitotic sister chromatid separation
negative regulation of mitotic sister chromatid segregation
mitotic DNA integrity checkpoint signaling
mitotic spindle checkpoint signaling
negative regulation of metaphase/anaphase transition of cell cycle
metaphase/anaphase transition of mitotic cell cycle
spindle assembly checkpoint signaling
regulation of spindle checkpoint
regulation of mitotic spindle checkpoint
negative regulation of mitotic sister chromatid separation
mitotic DNA damage checkpoint signaling
regulation of mitotic metaphase/anaphase transition
negative regulation of mitotic metaphase/anaphase transition
0 10 20 30 40
%Genes/Term
Summary term
Cell cycle process
regulation of cell cycle process
positive regulation of cell cycle
positive regulation of cell cycle process
positive regulation of mitotic cell cycle
positive regulation of cell cycle phase transition
positive regulation of mitotic cell cycle phase transition
DNA replication, synthesis of RNA primer
DNA-directed 5'-3' RNA polymerase activity
DNA primase activity 1
regulation of DNA-directed DNA polymerase activity 1
regulation of DNA primase activity 1
positive regulation of DNA primase activity 1
positive regulation of DNA-directed DNA polymerase activity 1
establishment of organelle localization
chromosome localization
establishment of chromosome localization
metaphase plate congression
mitotic sister chromatid segregation 1
mitotic metaphase plate congression
attachment of mitotic spindle microtubules to kinetochore
nuclear chromosome segregation
organelle organization
sister chromatid segregation
chromosome organization
regulation of chromosome organization
mitotic sister chromatid segregation 2
DNA conformation change 3
cytokinesis
regulation of cell division
positive regulation of cell division
cytoskeleton-dependent cytokinesis
mitotic cytokinesis
regulation of cytokinesis
positive regulation of cytokinesis
regulation of microtubule-based process
microtubule cytoskeleton organization
spindle organization
microtubule organizing center organization
cytoskeleton organization
centrosome cycle
microtubule cytoskeleton organization involved in mitosis
mitotic spindle organization
spindle assembly
non-membrane-bounded organelle assembly
cellular response to stress
cellular response to DNA damage stimulus
nucleic acid metabolic process
DNA metabolic process
DNA replication 2
regulation of DNA metabolic process
DNA repair
DNA recombination
recombinational repair
double-strand break repair
double-strand break repair via homologous recombination
DNA replication 3
DNA conformation change 4
DNA biosynthetic process
DNA-dependent DNA replication
regulation of DNA replication
DNA synthesis involved in DNA repair
DNA-directed 5'-3' RNA polymerase activity 2
DNA geometric change
DNA polymerase activity
DNA primase activity 2
regulation of DNA-dependent DNA replication
regulation of DNA-directed DNA polymerase activity 2
regulation of DNA primase activity 2
DNA-directed DNA polymerase activity
DNA unwinding involved in DNA replication
DNA duplex unwinding
positive regulation of DNA primase activity 2
positive regulation of DNA-directed DNA polymerase activity 2
0 25 50 75 100
%Genes/Term
Summary term
Cell response to DNA damage stimulus
Cytokinesis
DNA dependant replication
Mitotic sister chromatid segregation
Mitotic spindle organisation
Nuclear chromosome segregation
Regulation of cell cycle
Regulation of DNA polymerase activity
regulation of transferase activity
regulation of response to DNA damage stimulus
DNA replication initiation
cyclin-dependent protein serine/threonine kinase regulator activity
DNA-dependent DNA replication maintenance of fidelity
replication fork processing
cellular component organization
organelle organization
chromosome organization 4
attachment of spindle microtubules to kinetochore 1
regulation of attachment of spindle microtubules to kinetochore
attachment of mitotic spindle microtubules to kinetochore 1
DNA biosynthetic process
DNA synthesis involved in DNA repair
postreplication repair
cell cycle G1/S phase transition
G1/S transition of mitotic cell cycle
regulation of cell cycle G1/S phase transition
regulation of G1/S transition of mitotic cell cycle
DNA metabolic process 1
DNA replication 1
DNA-dependent DNA replication
regulation of DNA replication
microtubule cytoskeleton organization
microtubule organizing center organization
centrosome cycle
centrosome duplication
attachment of spindle microtubules to kinetochore 2
mitotic metaphase plate congression
attachment of mitotic spindle microtubules to kinetochore 2
protein localization to condensed chromosome
protein localization to kinetochore
mitotic cell cycle phase transition
cell cycle G2/M phase transition
G2/M transition of mitotic cell cycle
regulation of cell cycle G2/M phase transition
regulation of G2/M transition of mitotic cell cycle
chromosome organization 1
DNA conformation change 1
DNA packaging 1
DNA geometric change
DNA duplex unwinding
DNA conformation change 2
mitotic chromosome condensation
DNA packaging 2
regulation of chromosome condensation
chromosome condensation
positive regulation of chromosome condensation
cellular aromatic compound metabolic process
heterocycle metabolic process
organic cyclic compound metabolic process
nucleobase-containing compound metabolic process
nucleic acid metabolic process
DNA metabolic process 2
0 20 40 60 80
%Genes/Term
Summary term
Attachment of microtubules to kinetochore
Chromosome organization
DNA biosynthetic process
DNA conformation change
DNA metabolic process
DNA replication
DNA replication initiation
G1/S phase transition
Metaphase plate congression
Microtubule cytoskeleton organization
Mitotic phase transition
positive regulation of chromosome condensation
Protein serine/threonine kinase regulator activity
Regulation of transferase activity
Replication fork processing
Response to DNA damage stimulus

5B

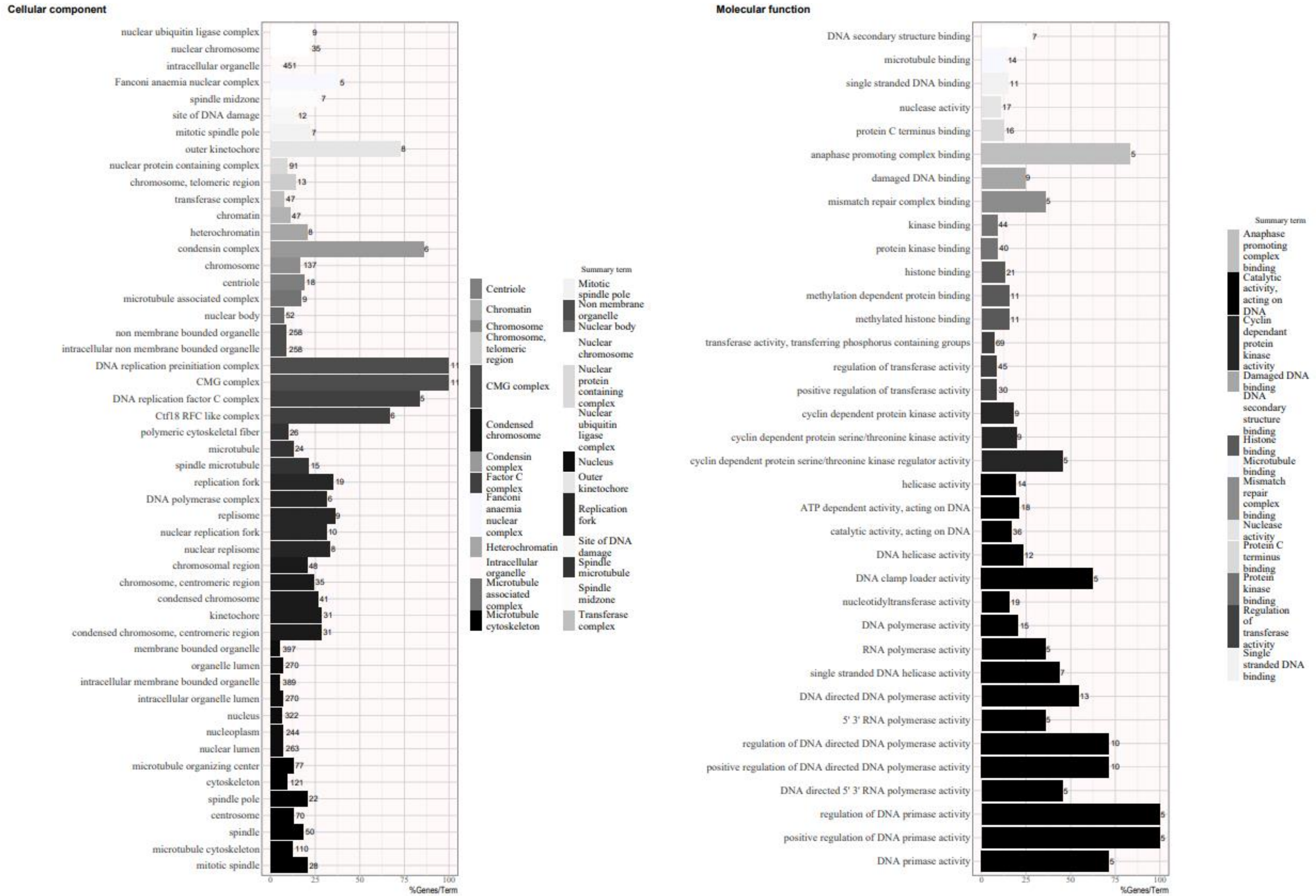
Cellular component
nuclear ubiquitin ligase complex
nuclear chromosome
intracellular organelle
Fanconi anaemia nuclear complex
spindle midzone
site of DNA damage
mitotic spindle pole
outer kinetochore
nuclear protein containing complex
chromosome, telomeric region
transferase complex
chromatin
heterochromatin
condensin complex
chromosome
centriole
microtubule associated complex
nuclear body
non membrane bounded organelle
intracellular non membrane bounded organelle
DNA replication preinitiation complex
CMG complex
DNA replication factor C complex
Ctf18 RFC like complex
polymeric cytoskeletal fiber
microtubule
spindle microtubule
replication fork
DNA polymerase complex
replisome
nuclear replication fork
nuclear replisome
chromosomal region
chromosome, centromeric region
condensed chromosome
kinetochore
condensed chromosome, centromeric region
membrane bounded organelle
organelle lumen
intracellular membrane bounded organelle
intracellular organelle lumen
nucleus
nucleoplasm
nuclear lumen
microtubule organizing center
cytoskeleton
spindle pole
centrosome
spindle
microtubule cytoskeleton
mitotic spindle
%Genes/Term
Summary term
Centriole
Chromatin
Chromosome
Chromosome, telomeric region
CMG complex
Condensed chromosome
Condensin complex
Factor C complex
Fanconi anaemia nuclear complex
Heterochromatin
Intracellular organelle
Microtubule associated complex
Microtubule cytoskeleton
Mitotic spindle pole
Non membrane organelle
Nuclear body
Nuclear chromosome
Nuclear protein containing complex
Nuclear ubiquitin ligase complex
Nucleus
Outer kinetochore
Replication fork
Site of DNA damage
Spindle microtubule
Spindle midzone
Transferase complex
Molecular function
DNA secondary structure binding
microtubule binding
single stranded DNA binding
nuclease activity
protein C terminus binding
anaphase promoting complex binding
damaged DNA binding
mismatch repair complex binding
kinase binding
protein kinase binding
histone binding
methylation dependent protein binding
methylated histone binding
transferase activity, transferring phosphorus containing groups
regulation of transferase activity
positive regulation of transferase activity
cyclin dependent protein kinase activity
cyclin dependent protein serine/threonine kinase activity
cyclin dependent protein serine/threonine kinase regulator activity
helicase activity
ATP dependent activity, acting on DNA
catalytic activity, acting on DNA
DNA helicase activity
DNA clamp loader activity
nucleotidyltransferase activity
DNA polymerase activity
RNA polymerase activity
single stranded DNA helicase activity
DNA directed DNA polymerase activity
5' 3' RNA polymerase activity
regulation of DNA directed DNA polymerase activity
positive regulation of DNA directed DNA polymerase activity
DNA directed 5' 3' RNA polymerase activity
regulation of DNA primase activity
positive regulation of DNA primase activity
DNA primase activity
%Genes/Term
Summary term
Anaphase promoting complex binding
Catalytic activity, acting on DNA
Cyclin dependant protein kinase activity
Damaged DNA binding
DNA secondary structure binding
Histone binding
Microtubule binding
Mismatch repair complex binding
Nuclease activity
Protein C terminus binding
Protein kinase binding
Regulation of transferase activity
Single stranded DNA binding

5C

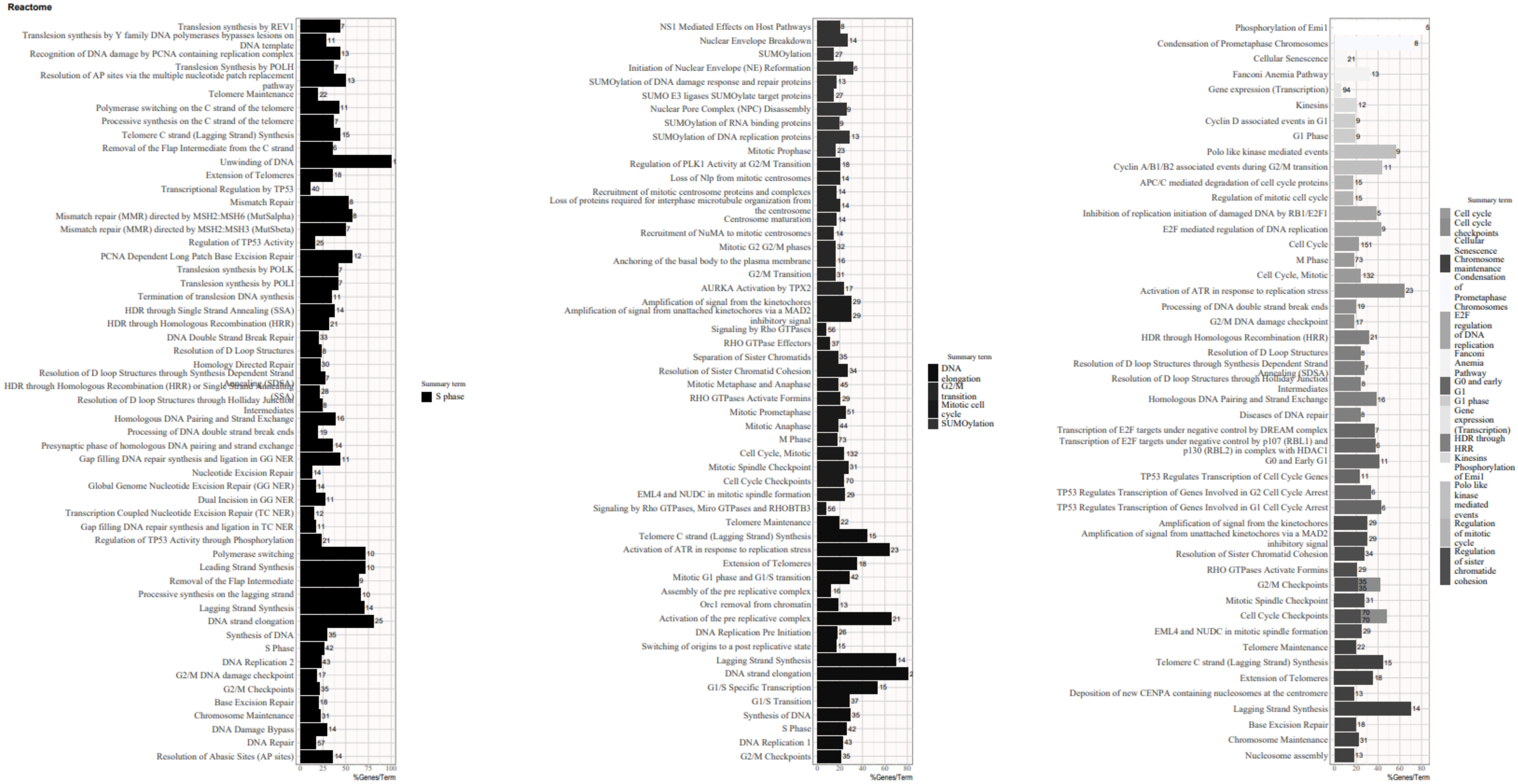


**Figure S6. Enrichment analysis of cluster 1 (Cell cycle process)** as given in Figure 6 which compares transcriptional profiles in 4 studied cell lines. Bar plots show gene sets and pathways of cluster 1. These were analyzed by A) biological process-, B) cellular component-, molecular function-gene ontology (GO) enrichment analysis and using C) Reactome pathway analysis. All significantly enriched terms as clustered into parental summary term by ClueGO (Cytoscape app) are given.

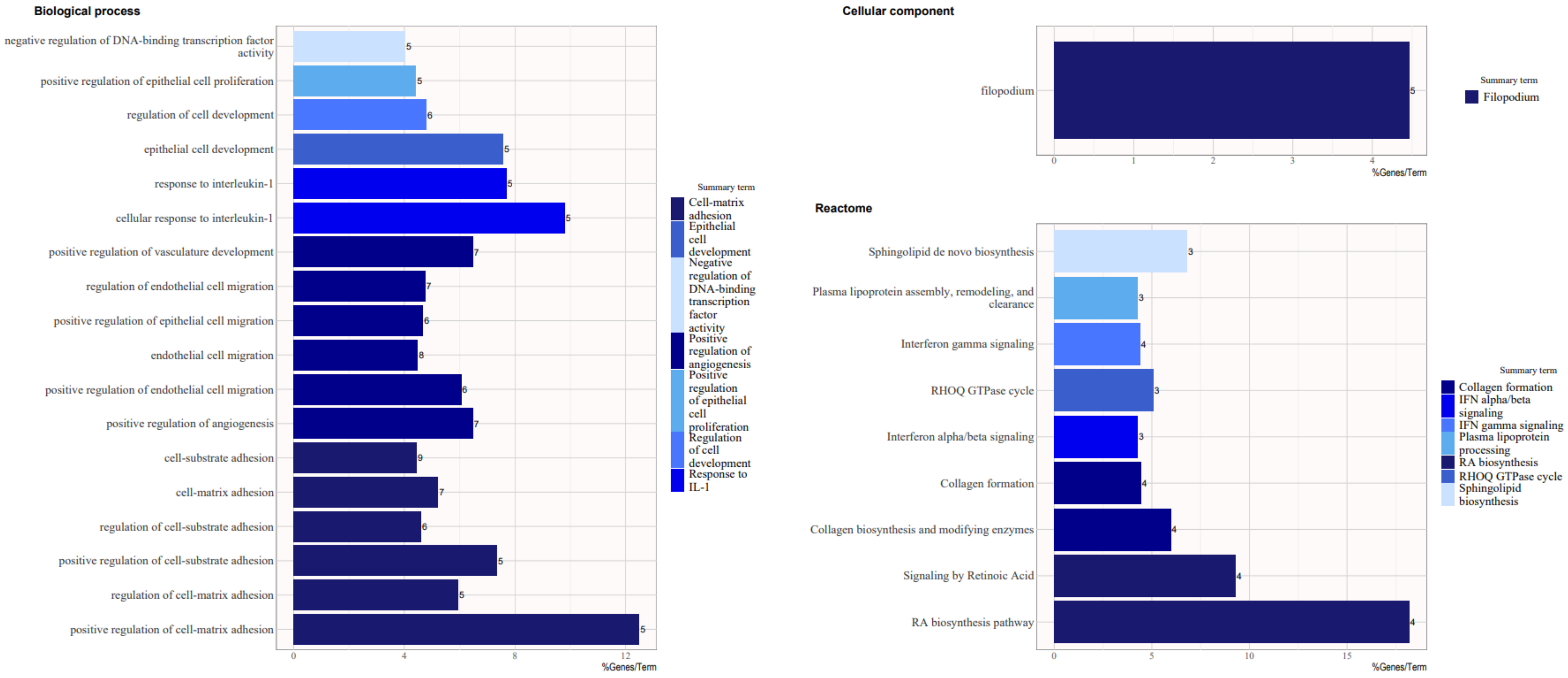


**Figure S7. Enrichment analysis of cluster 6 (Cell adhesion)** as given in Figure 6 which compares transcriptional profiles in 4 studied cell lines. Bar plots show gene sets and pathways of cluster 6. These were analyzed by biological process-, cellular component-, molecular function- gene ontology (GO) enrichment analysis and using Reactome pathway analysis. All significantly enriched terms as clustered into parental summary term by ClueGO (Cytoscape app) are given.

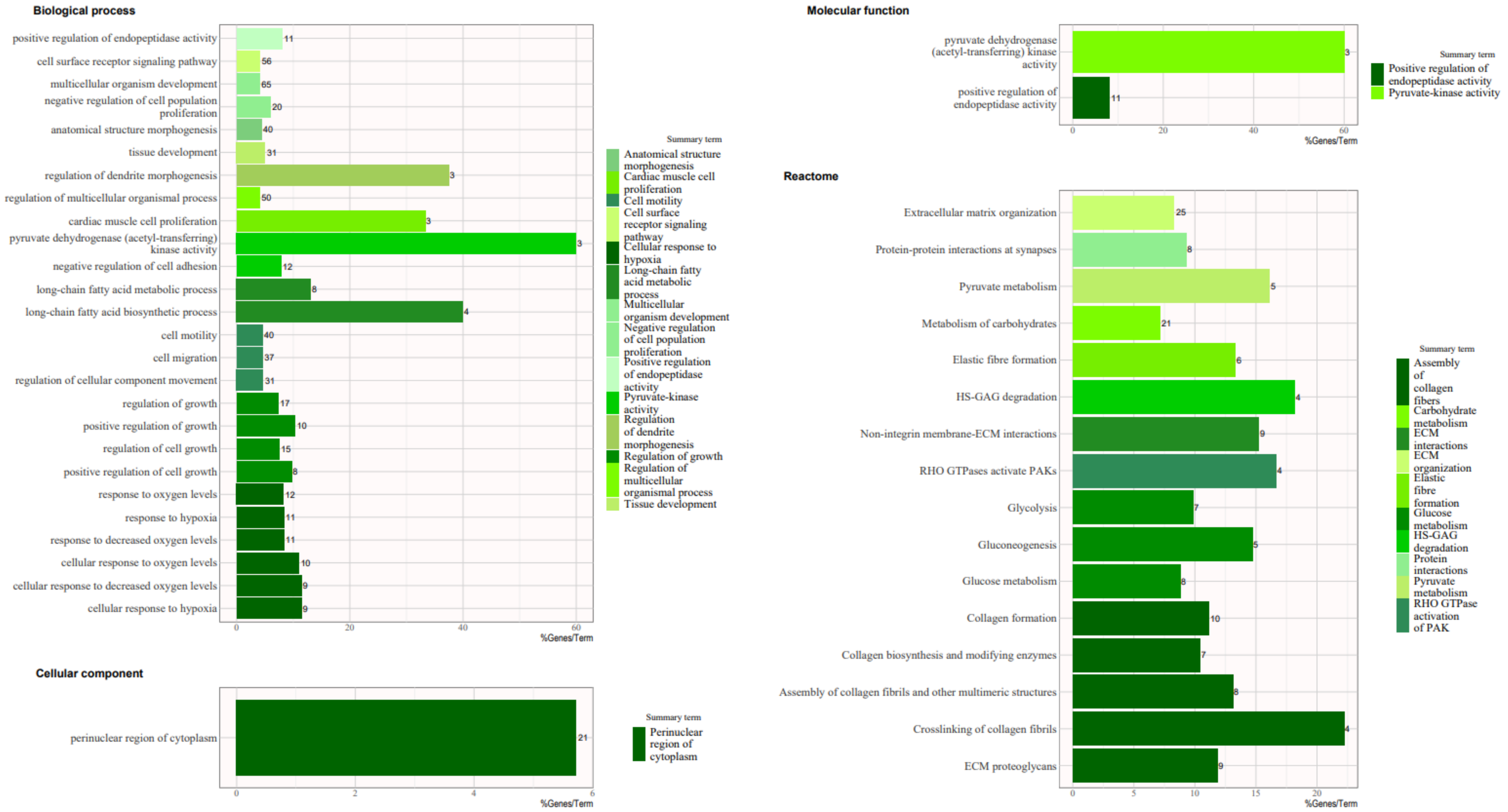


**Figure S8. Enrichment analysis of cluster 5 (Cell response to hypoxia)** as given in Figure 6 which compares transcriptional profiles in 4 studied cell lines. Bar plots show gene sets and pathways of cluster 5. These were analyzed by biological process-, cellular component-, molecular function-gene ontology (GO) enrichment analysis and using Reactome pathway analysis. All significantly enriched terms as clustered into parental summary term by ClueGO (Cytoscape app) are given.
