# Supplementary material for: Understanding Cell Model Characteristics—RNA Expression Profiling in Primary and Immortalized Human Mesothelial Cells, and in Human Vein and Microvascular Endothelial Cells
Source: Cells. 2022 Oct 5;11(19):3133. doi: 10.3390/cells11193133 (PMC9563025; doi:10.3390/cells11193133)
Supplement: Supplementary file 1 [file cells-11-03133-s001.zip › Supplemental figures_revised.pptx]

## Slide 1
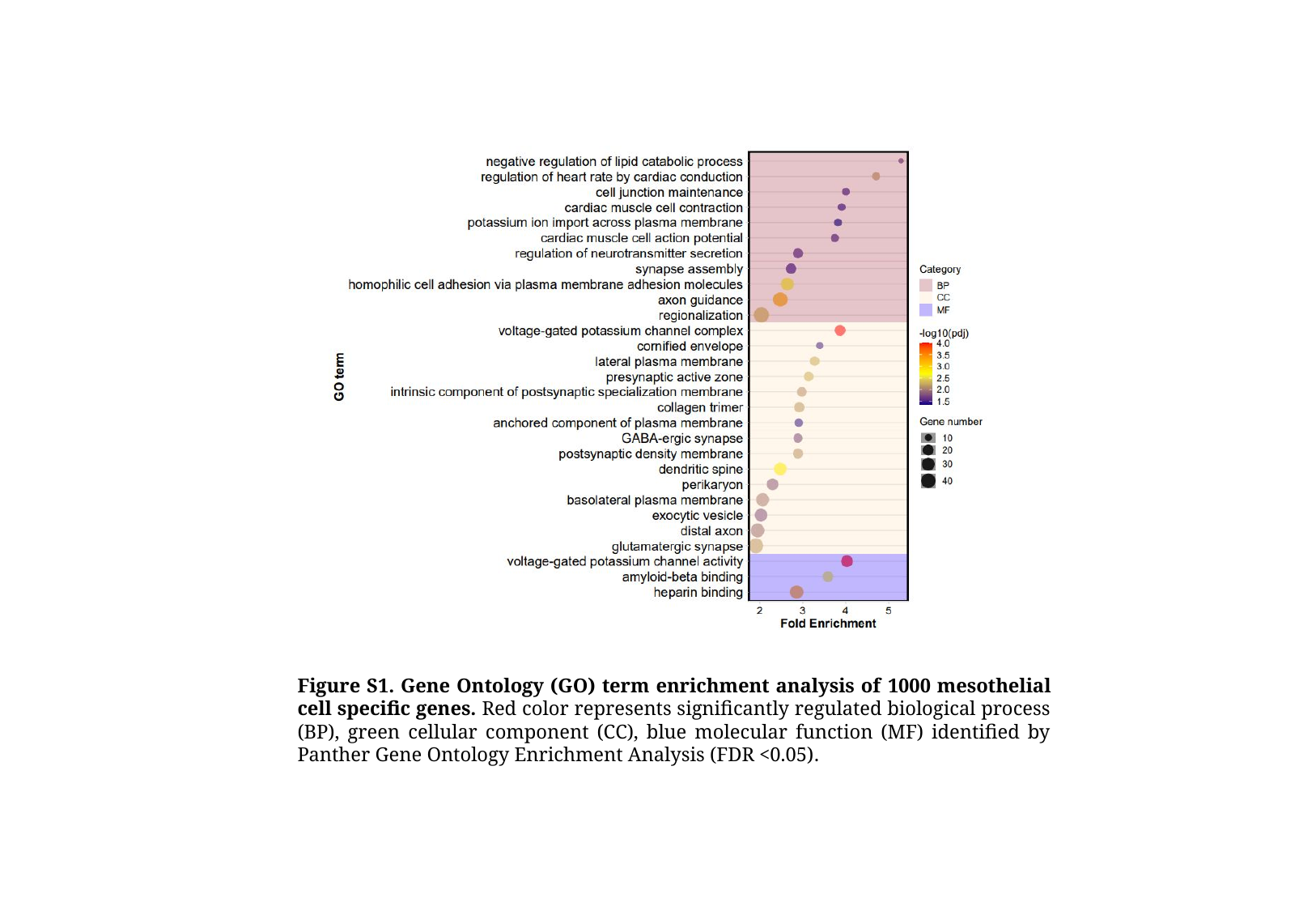

Figure S1. Gene Ontology (GO) term enrichment analysis of 1000 mesothelial cell specific genes. Red color represents significantly regulated biological process (BP), green cellular component (CC), blue molecular function (MF) identified by Panther Gene Ontology Enrichment Analysis (FDR <0.05).

## Slide 2
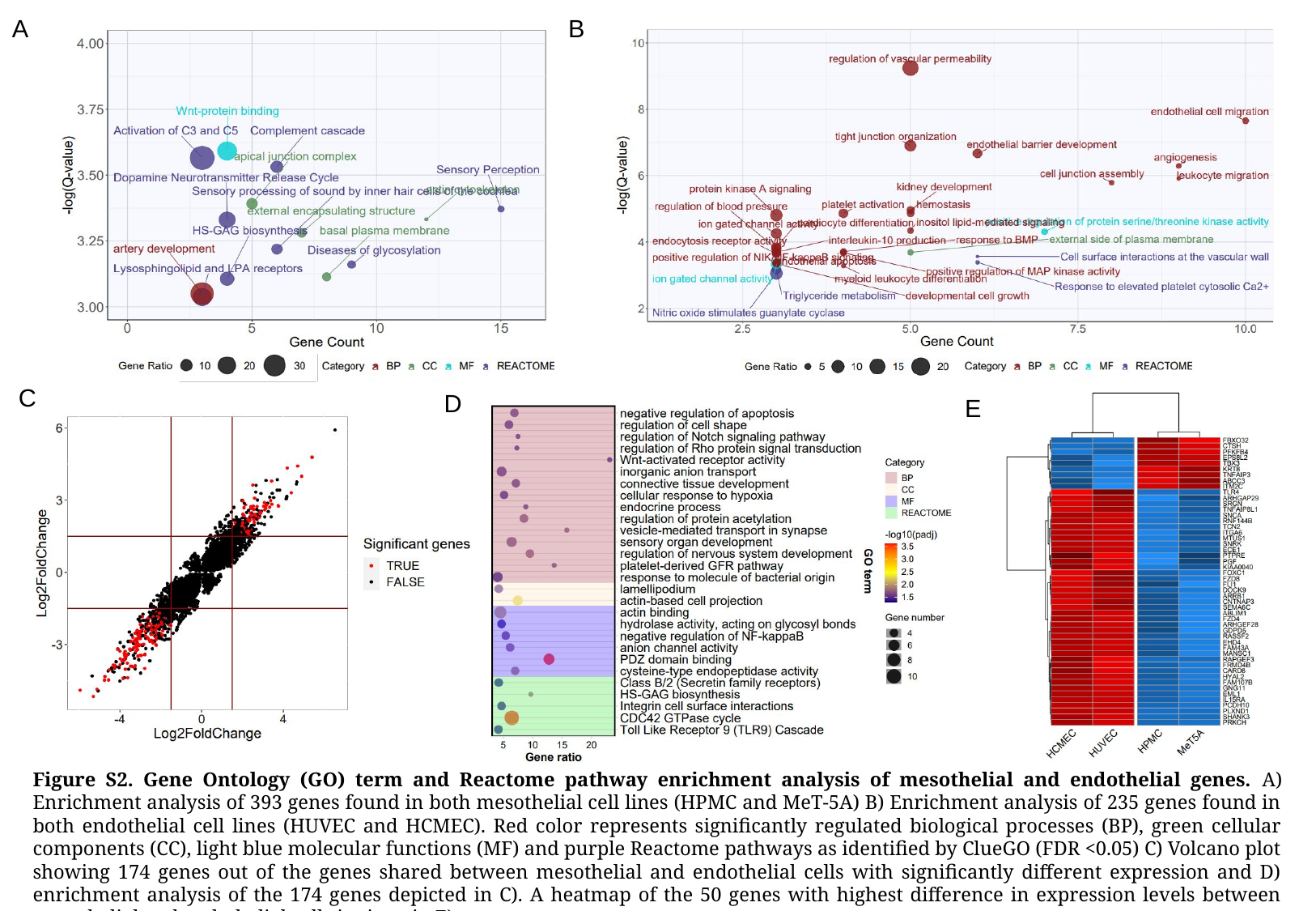

A
B
C
D
E
Figure S2. Gene Ontology (GO) term and Reactome pathway enrichment analysis of mesothelial and endothelial genes. A) Enrichment analysis of 393 genes found in both mesothelial cell lines (HPMC and MeT-5A) B) Enrichment analysis of 235 genes found in both endothelial cell lines (HUVEC and HCMEC). Red color represents significantly regulated biological processes (BP), green cellular components (CC), light blue molecular functions (MF) and purple Reactome pathways as identified by ClueGO (FDR <0.05) C) Volcano plot showing 174 genes out of the genes shared between mesothelial and endothelial cells with significantly different expression and D) enrichment analysis of the 174 genes depicted in C). A heatmap of the 50 genes with highest difference in expression levels between mesothelial and endothelial cells is given in E).

## Slide 3
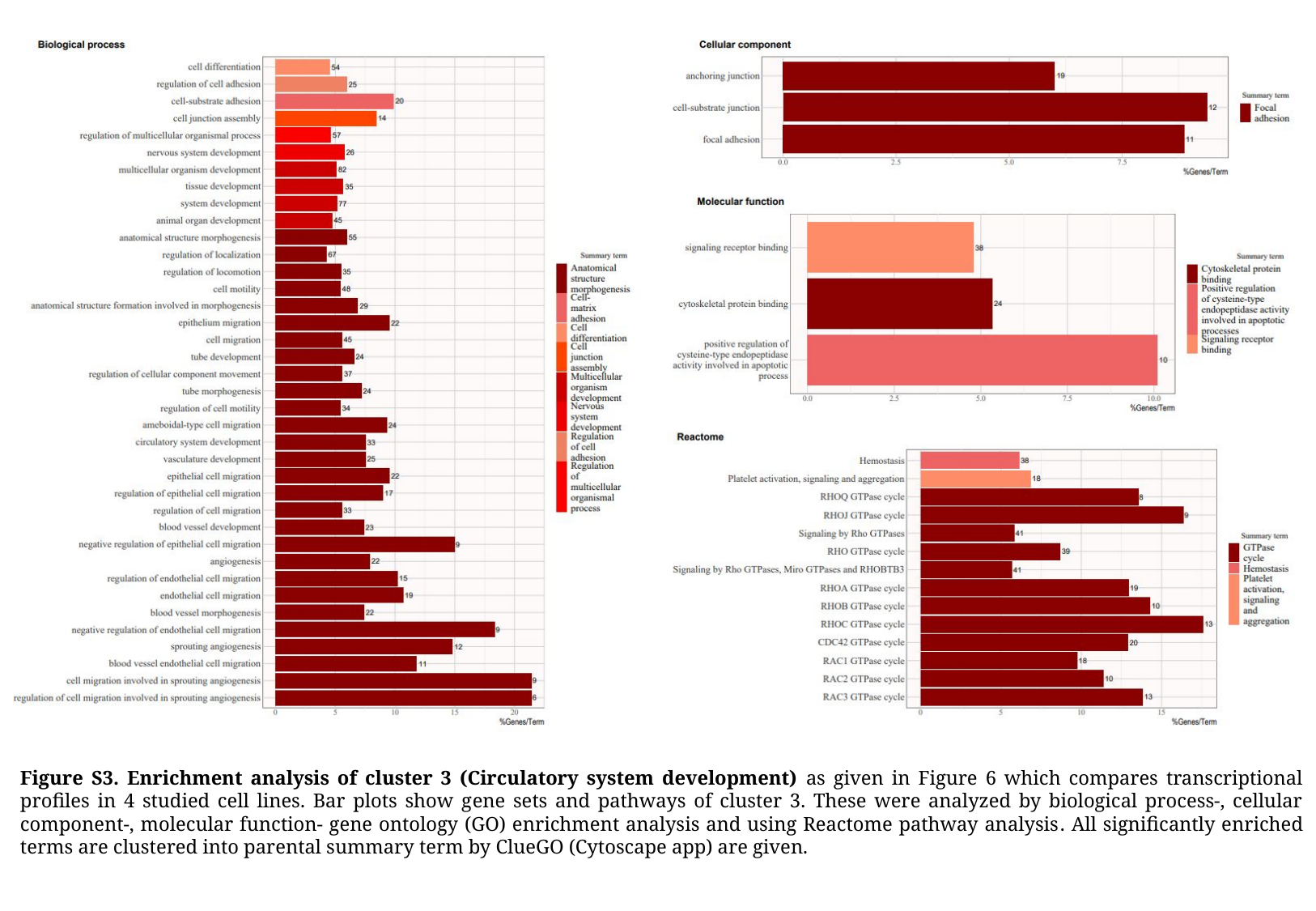

Figure S3. Enrichment analysis of cluster 3 (Circulatory system development) as given in Figure 6 which compares transcriptional profiles in 4 studied cell lines. Bar plots show gene sets and pathways of cluster 3. These were analyzed by biological process-, cellular component-, molecular function- gene ontology (GO) enrichment analysis and using Reactome pathway analysis. All significantly enriched terms are clustered into parental summary term by ClueGO (Cytoscape app) are given.

## Slide 4
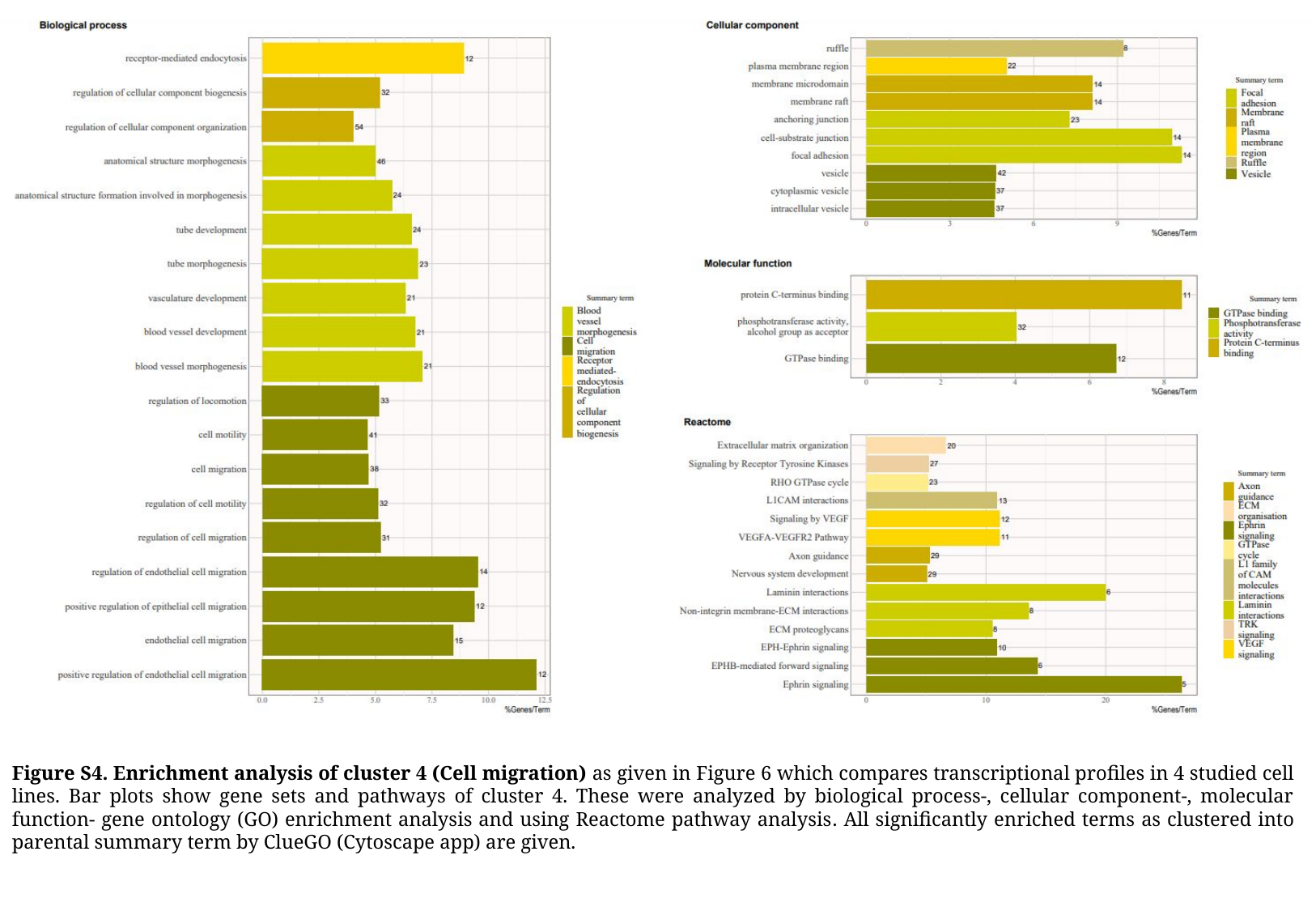

Figure S4. Enrichment analysis of cluster 4 (Cell migration) as given in Figure 6 which compares transcriptional profiles in 4 studied cell lines. Bar plots show gene sets and pathways of cluster 4. These were analyzed by biological process-, cellular component-, molecular function- gene ontology (GO) enrichment analysis and using Reactome pathway analysis. All significantly enriched terms as clustered into parental summary term by ClueGO (Cytoscape app) are given.

## Slide 5
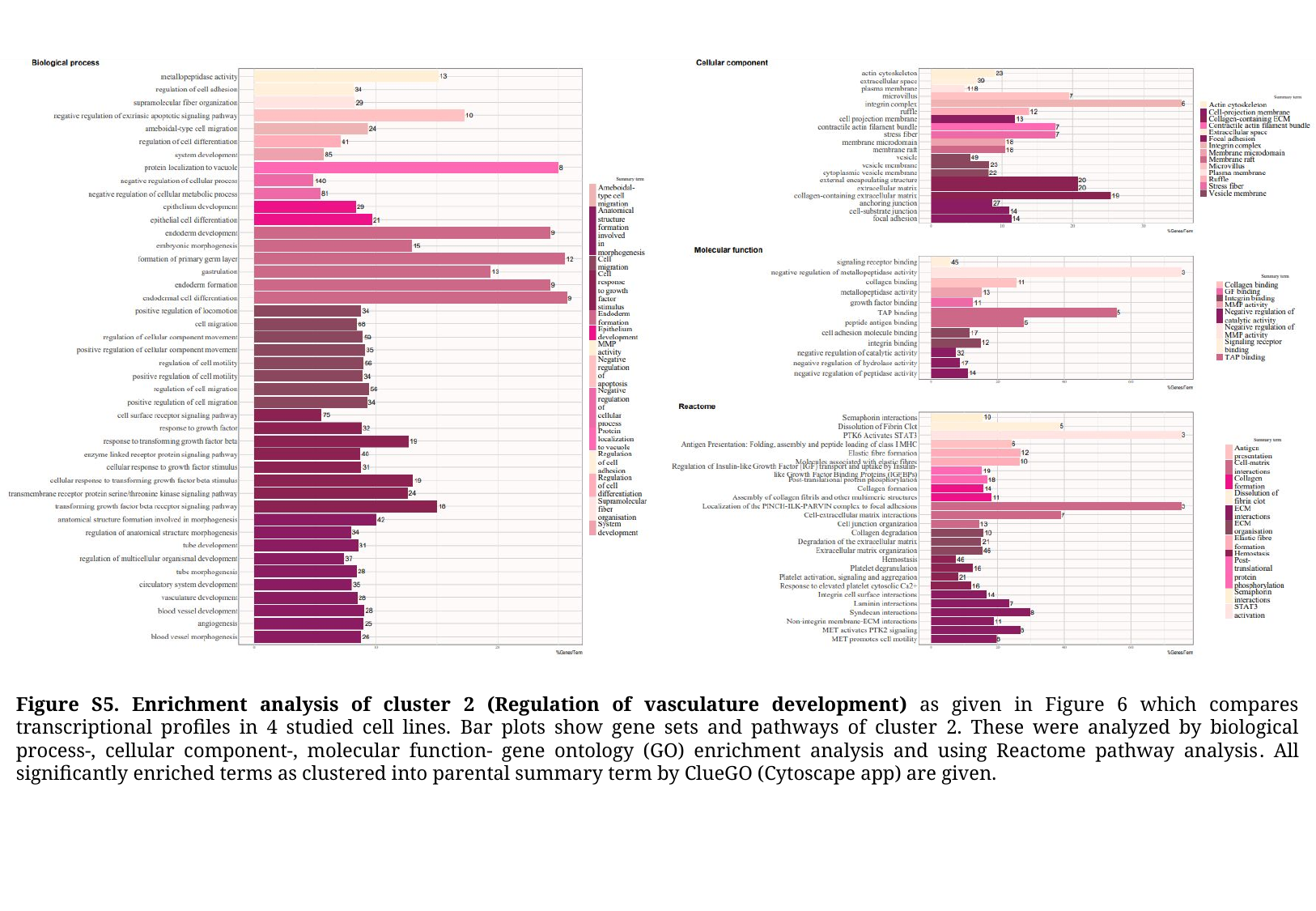

Figure S5. Enrichment analysis of cluster 2 (Regulation of vasculature development) as given in Figure 6 which compares transcriptional profiles in 4 studied cell lines. Bar plots show gene sets and pathways of cluster 2. These were analyzed by biological process-, cellular component-, molecular function- gene ontology (GO) enrichment analysis and using Reactome pathway analysis. All significantly enriched terms as clustered into parental summary term by ClueGO (Cytoscape app) are given.

## Slide 6
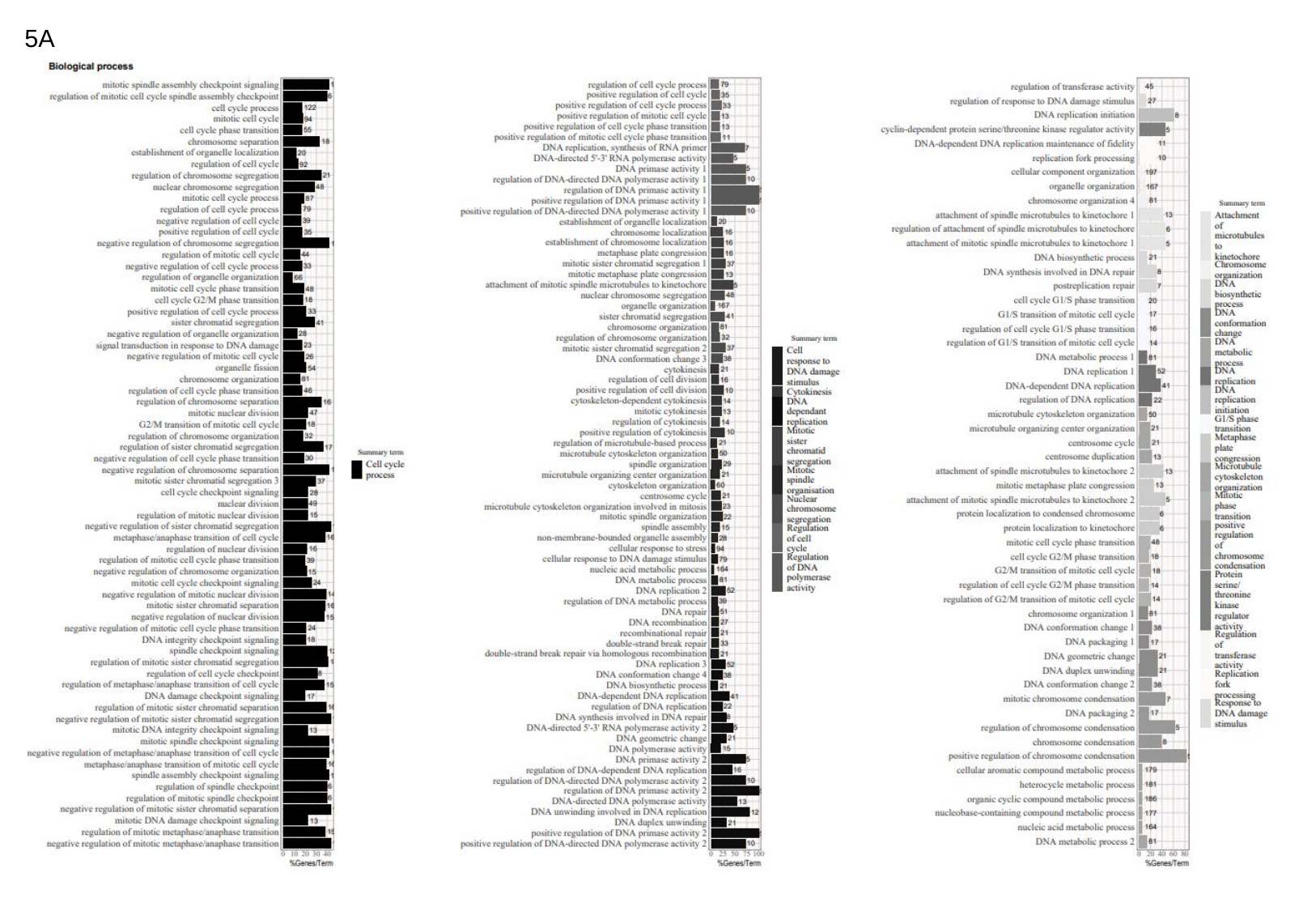

5A

## Slide 7
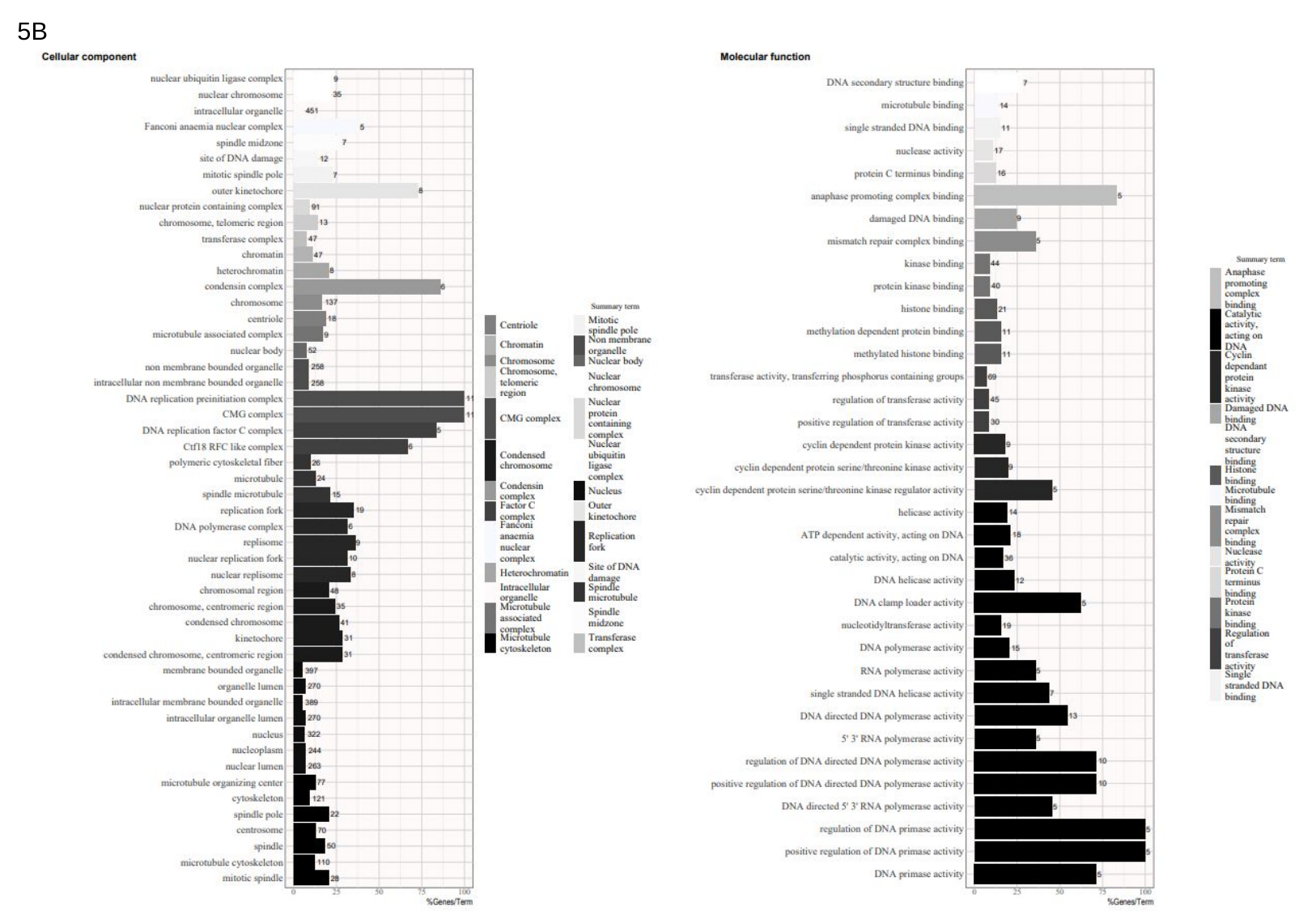

5B

## Slide 8
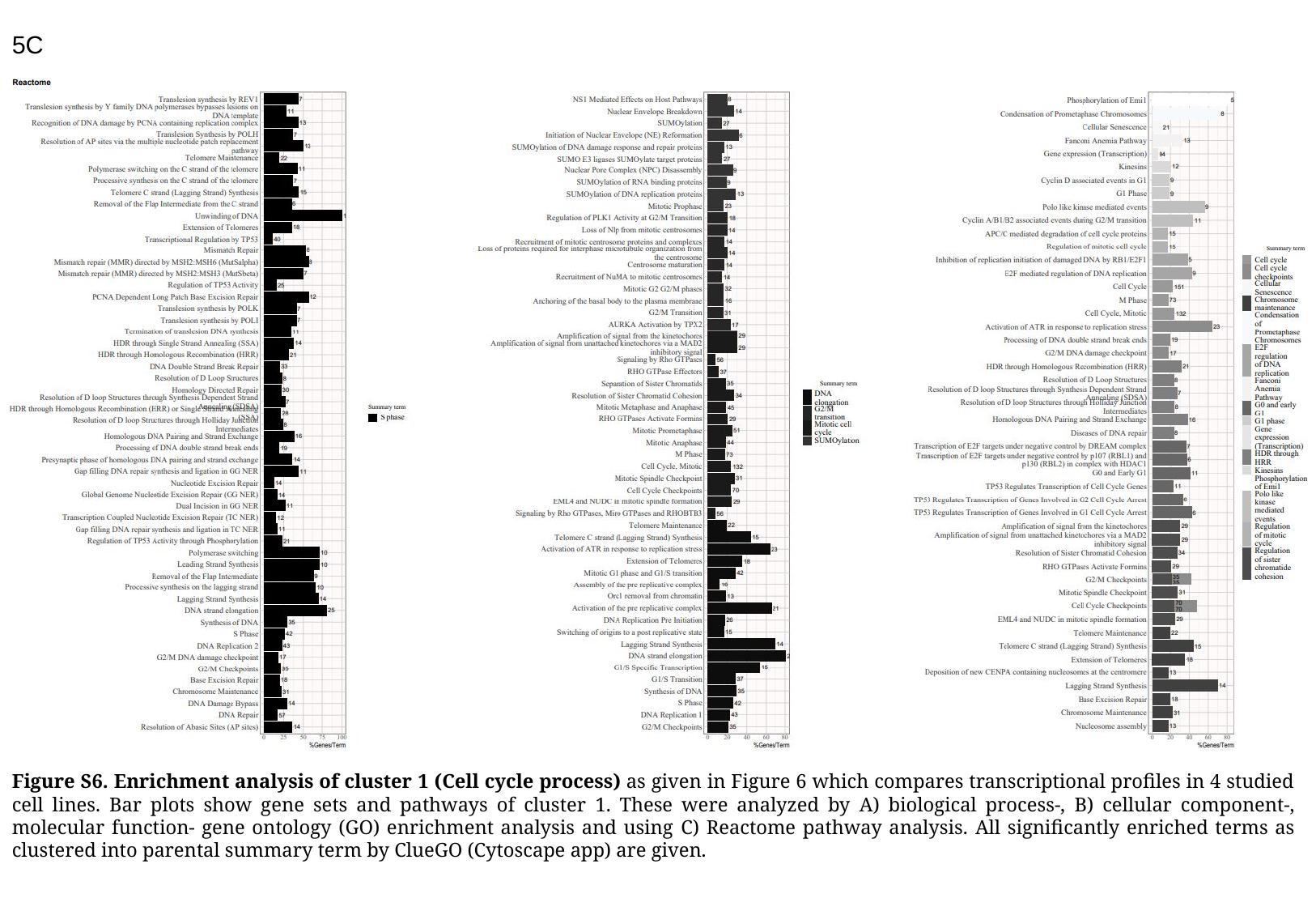

5C
Figure S6. Enrichment analysis of cluster 1 (Cell cycle process) as given in Figure 6 which compares transcriptional profiles in 4 studied cell lines. Bar plots show gene sets and pathways of cluster 1. These were analyzed by A) biological process-, B) cellular component-, molecular function- gene ontology (GO) enrichment analysis and using C) Reactome pathway analysis. All significantly enriched terms as clustered into parental summary term by ClueGO (Cytoscape app) are given.

## Slide 9
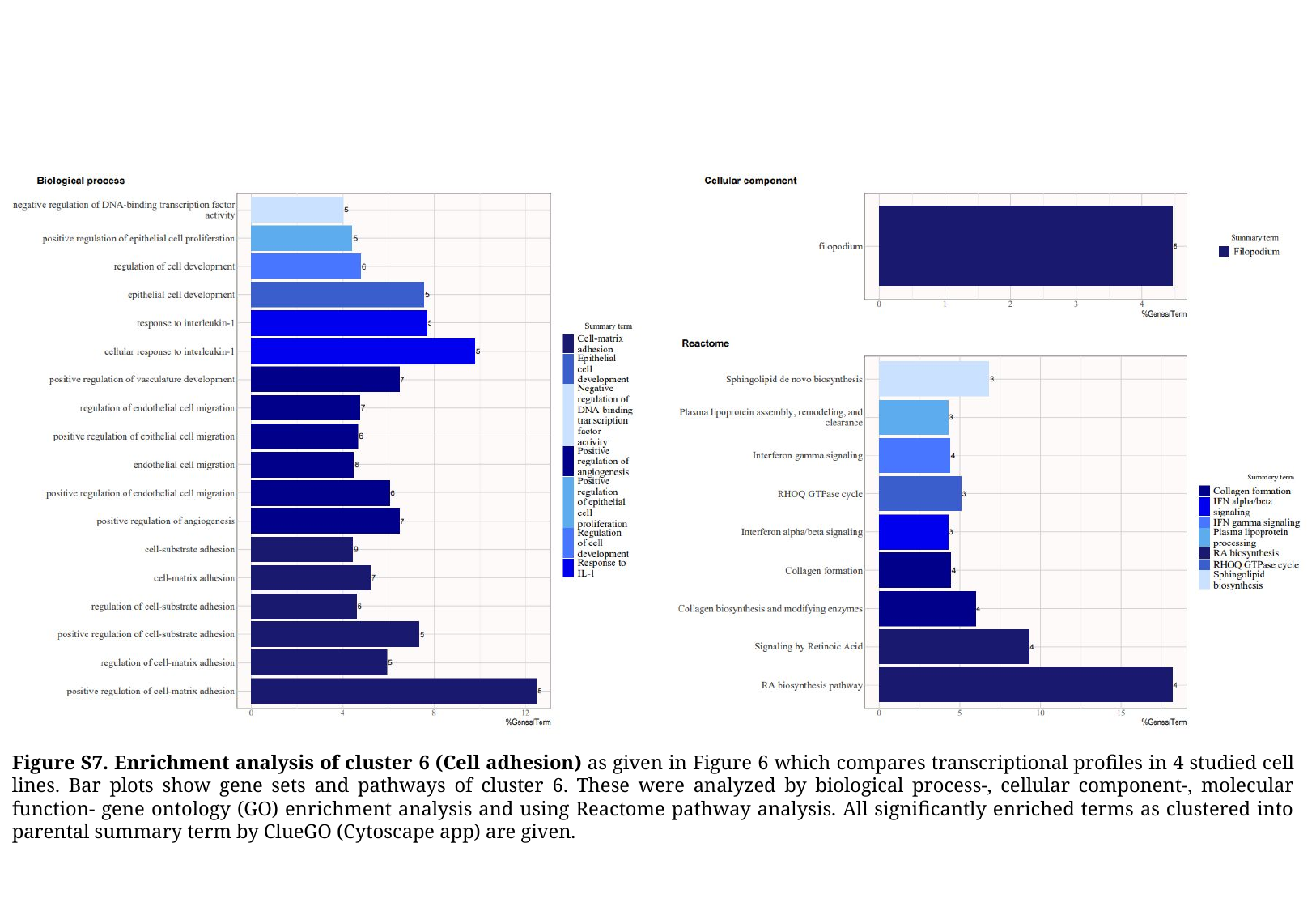

Figure S7. Enrichment analysis of cluster 6 (Cell adhesion) as given in Figure 6 which compares transcriptional profiles in 4 studied cell lines. Bar plots show gene sets and pathways of cluster 6. These were analyzed by biological process-, cellular component-, molecular function- gene ontology (GO) enrichment analysis and using Reactome pathway analysis. All significantly enriched terms as clustered into parental summary term by ClueGO (Cytoscape app) are given.

## Slide 10
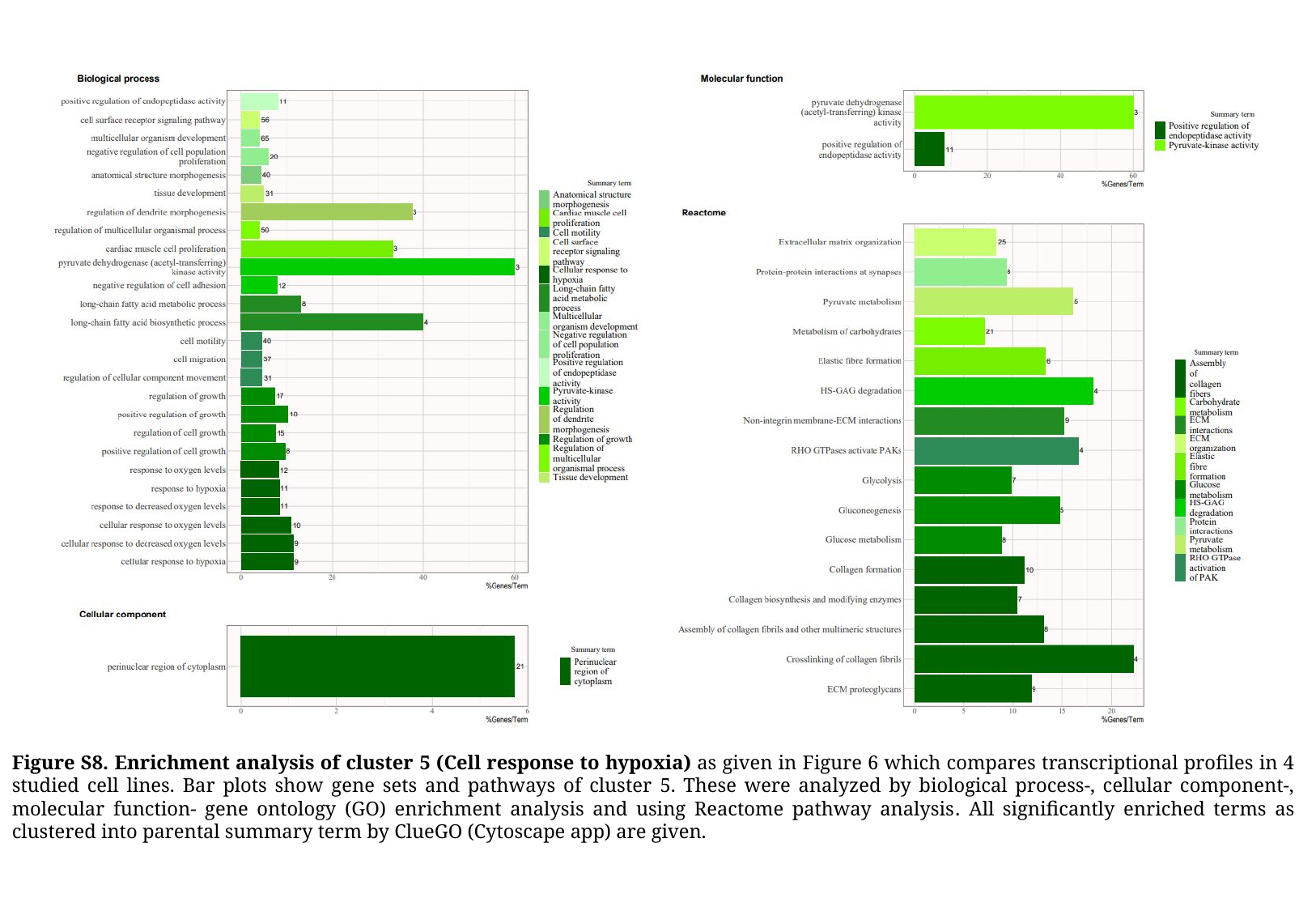

Figure S8. Enrichment analysis of cluster 5 (Cell response to hypoxia) as given in Figure 6 which compares transcriptional profiles in 4 studied cell lines. Bar plots show gene sets and pathways of cluster 5. These were analyzed by biological process-, cellular component-, molecular function- gene ontology (GO) enrichment analysis and using Reactome pathway analysis. All significantly enriched terms as clustered into parental summary term by ClueGO (Cytoscape app) are given.
